# Effects of mealworm larva composition and selected process parameters on the physicochemical properties of extruded meat analog

**DOI:** 10.1002/fsn3.2414

**Published:** 2021-06-25

**Authors:** Sun Young Cho, Gi Hyung Ryu

**Affiliations:** ^1^ Department of Food Science and Technology Food and Feed Extrusion Research Center Kongju National University Yesan Korea

**Keywords:** die temperature, extruded meat analog, extrusion, mealworm larva, moisture content

## Abstract

Mealworm larva (*Tenebrio molitor*), the most promising edible insect species with low cost and less environmental pollution, can fulfill the flavor and nutrition which are deficient in soy‐based meat analog. Consumers who might have the sensitivity and reluctance to the intact form of edible insect could be offered the high‐quality extruded meat analog. Therefore, this study aimed to investigate the effects of different mealworm larva contents and extrusion process parameters using twin‐screw extruder on the physicochemical properties of the extruded meat analog. Mealworm larva was added to the base formulation at the rate of 0, 15, and 30%. Extrusion process parameters were varied as die temperature (140°C and 150°C) and moisture content (40% and 50%). The integrity index, texture profile analysis, and oxidation activity (TBARS) of extruded meat analog decreased with increase in mealworm larva content. However, water holding capacity, nitrogen solubility index, protein digestibility, and DPPH radical scavenging activity significantly increased (*p* < .05) as the mealworm content increased. Lower die temperature and higher moisture content enhanced the textural properties, but reduced nitrogen solubility index and protein digestibility. At higher die temperature, DPPH activity increased but TBARS showed the opposite result. After extrusion cooking, the total amino acid levels of extruded meat analog were 585.21 g/kg in 30% mealworm larva content that level was lower than 591.43 g/kg in raw mixture while the sulfur‐containing amino acids and glutamic acid were higher than that of raw mixture. In conclusion, addition of mealworm larva could improve the nutritional value, antioxidant functionality, and taste of extruded meat analog under controlled extrusion process conditions.

## Graphical abstract

Antioxidant activity is an important factor in the presented study. Especially, we confirmed that mealworm larva possesses antioxidant activity whereas oxidation substance detects low content. TBARS (rancidity) was influenced by increasing the antioxidant property (containing mealworm larva).

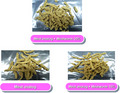

## 1 INTRODUCTION

The meat production has various health and environmental issues, and the global consumers are being aware of these concerns for their well‐being and health. Hence, the demand for plant‐based meat analog as an alternative food is increasing (Cho & Ryu, 2017). Meat analog texturized from plant proteins by means of extrusion technology contains highly beneficial essential amino acids, low saturated fat, no cholesterol, and fiber (Samard et al., 2019). It has year‐long shelf stability in dry form and capacity for rehydration without loss in structure, shape, and chewy texture (Harper, 1981). The major component of meat analog is protein (50–95%, dry matter). Soy protein is a major important component to create the structure of meat analog. The nutritional value of soy protein is comparable to that of meat protein. It contains relatively well‐balanced composition of amino acids (Ma & Ryu, 2019).

Extrusion cooking is presently the principal processing technology used for texturization of meat analogs. It can create the meat‐like cell structure from soy protein by means of high moisture, temperature, and shear. It can also eliminate the bitter taste and odor. There are also industrial advantages in terms of cost‐effectiveness and the capability for producing large amount in short time (Harper, 1981). Extrusion cooking using high moisture content (40%–80%) could produce highly fibrous meat analog comparable to meat, poultry, or fish muscle (Lin et al., 2002). However, consumers generally judge the overall sensory quality of meat analog to be lower than that of meat (Neville et al., 2017). The meat analog exhibits weak flavor and is almost tasteless, which has resulted in limited market success. For consumer acceptance, the quality of meat analog can be improved by incorporating animal protein into formulation which generally enhances flavor, sensory, texture, and nutrition values of final products (Liu et al., 2005). By addition of animal protein to meat analog, the use of excessive seasoning, flavoring, and coloring can also be omitted (Cho & Ryu, 2017a). Chiang et al. (2020) reported that addition of Maillard‐reacted beef bone by‐products enhanced the organoleptic properties of meat analog.

Furthermore, edible insects contain soy‐deficient essential fatty acids and essential amino acids such as methionine. They contain high‐quality proteins, fats, vitamins, and various minerals when compared to beef and pork. Edible insects are alternative sustainable sources for animal protein. They are also considered as tasty and even delicious by some consumers (Defoliart, 1999). The high‐quality protein in edible insect has also played an important role in reducing Kwashiorkor in young children of Africa (Defoliart, 1999). Moreover, they are food materials that could be produced at low cost without polluting the environment (Siemianowska et al., 2013). There is evidence that edible insects emit less greenhouse gases and ammonia, and thus, they are considered as more environmentally friendly compared to conventional livestock (Oonincx et al., 2010). Therefore, they are being considered as valuable food and feed source and can contribute to the increasing demand for animal proteins along with the increasing world population. The food and agriculture organization of UN predicted that the human population will be increased up to 9 billion by 2050 (Siemianowska et al., 2013). It may lead to shortages of food sources, especially animal protein. Hence, edible insects present alternative to animal protein in the future.

Among edible insects, mealworm larva (*Tenebrio moiltor*) contains high protein and fat while its carbohydrate content is relatively lower than that of other edible insects (Rumpold & Schlüter, 2015). Unsaturated fatty acids such as omega‐3 and omega‐6 are found in large quantities in mealworm larva, and they are as high as in fish (Van Huis, 2013). In a feasibility study for inclusion of insects in poultry diet, mealworm larva has been identified as the most promising species for mass‐rearing on an industrial scale in the western world (Rumpold & Schlüter, 2015).

On the other hand, consumers in some countries are still sensitive to consume edible insects in their intact form (Chung, 2010). In promoting the entomophagy, there are some serious issues including disgust and sensitivity, beliefs about the risk of consuming insects, and reluctance to try new foods. It seems that people might accept eating insects if they do not see the original form. Therefore, the conversion of mealworm larva into flour form and mixing into the formulation of meat analog might improve the flavor, nutritional quality, and functionality of meat analog and also reduce the consumers' disgust of insect's intact form.

Previous studies have been focused on incorporating chicken (Megard et al., 1985), pork (Liu et al., 2005), tuna sawdust (Cho & Ryu, 2017a), and beef bone hydrolysate (Chiang et al., 2020) into extruded meat analog. However, there has been no study about the addition of mealworm into meat analog under various extrusion process parameters. The texture of meat analog originally depends on the extrusion process parameters including moisture content and die temperature. Therefore, the objective of the study was to investigate the effects of die temperature (140°C and 150°C) and moisture content (40% and 50%) on physicochemical properties of meat analog extruded with addition of different levels of mealworm (0%, 15%, and 30% dry basis). The textural properties (hardness, cohesiveness, springiness, and chewiness, and water holding capacity, integrity index, nitrogen solubility index), the nutritional value (total amino acids, protein digestibility), and functionality (antioxidant property, lipid rancidity) of extruded meat analog were studied.

## 2 MATERIALS AND METHODS

### 2.1 Materials

Defatted soy flour, isolated soy protein, and corn starch were purchased from Samchang, Solae Co., and Roquette Freres, respectively. Roll mill (Shinpoong Eng., LTD) was used to grind the dehydrated mealworm larva (*Tenebrio molitor*, M.G Natural). Ninhydrin (Duksan Chemical Co.), ethylene glycol (Daejung Chemical Co.), acetic acid (Daejung Chemical Co.), sodium acetate (Duksan Chemical Co.), stannous chloride II (Kokusan Chemical Co., Kitasaiwai Nishi‐ku). Pepsin from porcine gastric mucosa (powder, ≥250 unit/mg solid), 2,2‐diphenyl‐1‐picrylhydrazyl, and 2‐thiobarbituric acid were purchased form Sigma‐Aldrich, Seoul, Korea. All other reagents and chemicals used were of analytical grade.

### 2.2 Proximate composition

Measurements were done according to American Association of Cereal Chemists (1999). Moisture content was measured by drying the samples at 105°C. Crude fat content was measured by Soxhlet extraction. Crude protein was determined by ninhydrin method (Starcher, 2001).

### 2.3 Extrusion conditions of raw samples

A co‐rotating intermeshing twin‐screw extruder (Incheon Machinery Co., Ltd.) with measurement of 32 mm diameter and 768 mm length (L/D ratio = 24) equipped with a rectangular die (10 mm width and 4.5 mm high) was used. This study adapted the base mixture reported by Cho and Ryu (2017a) which was composed of 65% defatted soy flour (DSF), 25% isolated soy protein (ISP), and 10% corn starch (w/w). In this base mixture, mealworm larva was added at the ratio of 100:0, 85:15, and 70:30 (base mixture: mealworm larva (w/w)). The mixtures were passed through the sieve of 60 mesh at room temperature.

Extrusion conditions for texturization of meat analog with different mealworm larva contents were varied for die temperature (140°C and 150°C) and moisture content (40 and 50%). Feed rate and screw speed were set at 100 g/min and 250 rpm and barrel temperatures of 100°C at feed, 160°C at compression, and 130°C at metering sections, respectively. After extrusion, the samples of 1 × 0.45 × 8 cm (W × H × L) were dried for 8 hr at 50°C, and then, the physical properties were determined using pieces of cutting samples (1 × 1 × 1 cm). The chemical properties were measured by using ground particles from stainless steel mixer (FM‐909T, Hanil, Haman) that have passed through 14–30 mesh (US Standard Sieve Series).

### 2.4 Texture profile analysis of extruded samples

Texture profile analysis (TPA) was determined with Sun rheometer (Compac‐100 Ⅱ, Sun Sci. Co.) equipped with a 10‐kg load cell and a cylinder probe of 25 mm in diameter. The dimensions (1 × 1 × 1 cm) of each sample were measured with Vernier caliper (CD‐15CPX, Mitutoyo Co., Ltd.). Before measurement, the samples were hydrated at 100°C for 20 min. The hardness, cohesiveness, springiness, and chewiness were measured according to the analysis method described by Samard et al. (2019). As measurement conditions, the samples were compressed to 75% of its original height (10 mm) at a cross‐head speed of 60 mm/min with the distance between two supports of 13 mm, the angle of probe type of 65°, and the maximum peak stress of 10 kg. Determinations were based on ten measurements and then were calculated as described by Breene (1975).

### 2.5 Integrity index of extruded samples

Integrity index was determined by modified method of Cho and Ryu (2017a). Dried sample (5 g) was placed in conical flask containing 100 ml of distilled water and then soaked at 80°C. After 30 min, the sample was placed in a PAC‐60 autoclave (Lab House Co., Ltd.) at 121°C for 30 min and transferred again into 100 ml distilled water to cool and was homogenized by homogenizer (IKA Co., Ltd.) for 1 min at 25,270 × *g*. Then, it was drained through the sieve of 20 mesh and dried at 80°C for 2 hr. The integrity index in percent was calculated as the residue weight divided by the dried sample weight.

### 2.6 Water holding capacity of extruded samples

Water holding capacity (WHC) was determined by method of Lin et al. (2002). Approximately 25 g of dry sample was weighed and rehydrated at 50°C in the distilled water for 12 hr followed by draining for 5 min. The water holding capacity was calculated by subtracting dry sample weight from wet sample weight and divided by dry sample weight.

### 2.7 Total amino acids in raw mixture and extruded samples

Total amino acids were assayed using ion‐exchange chromatography method modified from Association of Official Analytical Chemists (2005). Sample (0.2 g) was put into the digestion tube, added with 40 ml of 6 N HCL, and hydrolyzed for 24 hr at 110°C after the injection of nitrogen gas. The filtrate was concentrated with decompression concentrator (CCA‐1111‐CE, EYELA, Unit C Bohemia).

### 2.8 Nitrogen solubility index of extruded samples

Nitrogen solubility index (NSI) was determined by the modified method of Cho and Ryu. (2017a). Sample (1.5 g) was put into 75 ml of KOH solution and was stirred in a shaker (SI‐300R, Jeiotech) at 200 rpm. Solution (50 ml) was centrifuged at 1,800 × *g* for 30 min to acquire 0.5 ml of the supernatant. Soluble nitrogen content was measured by using ninhydrin method (Starcher, 2001). The total nitrogen content was measured by completely hydrolyzing 1.5 g of the sample in 6 N HCL for 24 hr at 100°C. The hydrolysates were dissolved in 75 ml of distilled water. The 0.5 ml of the supernatant was collected, and it was measured by using ninhydrin method (Starcher, 2001). NSI was determined with the following equation:

(1) NSI (%)=Soluble nitrogen content/Total nitrogen content in sample×100

### 2.9 Protein digestibility of extruded samples

Protein digestibility (PD) was determined by modified method of Mertz et al. (1984). The samples (200 mg) were suspended in 35 ml of pepsin solution (1.5 g of enzyme/1,000 ml of 0.084 N HCL). These samples were digested at 37°C for 2 hr in a shaker by stirring at 150 rpm. After digestion, 2 ml of 2 M NaOH was added to stop enzyme activity. These solutions were centrifuged at 1,800 × *g* for 15 min. The supernatant was decanted, and the residue was washed with 10 ml of 0.1 M phosphate buffer. The residues were washed for two more times. The undigested residues were dried at 30°C for 2 days. Undigested protein content of these samples was analyzed using ninhydrin method (Starcher, 2001). The protein digestibility percentage was determined with the following equation:

(2) PD (%)=Total protein‐Undigested protein/Total protein content in sample×100

### 2.10 DPPH radical scavenging activity of extruded samples

The DPPH radical scavenging activity was determined according to the method of Brand‐Williams et al. (1995). The extraction of sample (1 g) was done with 80% methanol at room temperature for 2 hr and centrifuged at 1,800 × *g* for 30 min. The DPPH solution was prepared by adding 0.0024 g of DPPH reagent to 100 ml of methanol, and the solution (3.9 ml) was added to 0.1 ml of supernatant. After incubation at room temperature for 30 min in the dark, the absorbance was read at 515 nm using spectrophotometer (Biowave 3, Biochrom WPA, Co., Ltd.). The DPPH radical scavenging percentage was determined with the following equation:

(3) $$\text{DPPH radical scavenging activity}\;\left(\%\right)=\left[\left({\text{Abs}}_{\text{control}}‐{\text{Abs}}_{\text{sample}}\right)/{\text{Abs}}_{\text{control}}\right]\times 100$$

where Abs_control_ is the absorbance of the control and Abs_sample_ is the absorbance of the sample.

### 2.11 TBARS of extruded samples

The TBARS value was determined by the method of Pyun et al. (2012). The sample (1 g) was mixed with 45 ml of 0.3 M HClO_4_ and 0.3 ml of 3% BHT solution dispersed in ethanol. The sample was extracted at room temperature for 2 hr and filtered using the filter paper. The 5 ml of filtrate was collected in a glass tube. The TBA reagent (5 ml) obtained by mixing 0.69% of 2‐thiobarbituric acid and 99.5% glacial acetic was added to the filtrate in the glass tube. After boiling it at 100°C for 35 min and cooling, the absorbance was read at 531 nm using spectrophotometer (Biowave 3, Biochrom WPA, Co., Ltd.). The malonaldehyde content per kg was determined by using the following equation:

(4) $$\text{TBARS}\;\left(\mu g/g\right)=\left({\text{Abs}}_{\text{sample}}‐{\text{Abs}}_{\text{control}}\right)\times 3\times 100/S$$

where Abs_sample_ is the absorbance of the sample, Abs_control_ is the absorbance of the control, and S is weight of sample (g).

### 2.12 Sensory evaluation of extruded samples

For sensory evaluation, the dry samples were ground passing through a meat chopper (KM7, Samwoosa Co.) and drained on a US standard 8‐mesh sieve for 10 min. The samples (5 g) were dispensed into plastic cups with 3‐digit codes on the sides and served to 10 panelists of trained judges in the meat analog industry (researchers of 6 men and 4 women, who were between 23 and 40 years old). Sensory analysis was done by the panelists with given description of five‐point scale referring to El‐Refai et al. (2014). The five‐point scale used was as follows: (5) excellent, (4) good, (3) medium, (2) poor, and (1) very poor. A total of 5 evaluation sessions was conducted with drinking water for mouth rinsing between samples.

### 2.13 Statistical analysis

All experiments were performed in triplicate. Analysis of variance (ANOVA) and mean values were compared by using Duncan's multiple range tests at 5% level of significance. All the statistical analyses were conducted using the SPSS for Window 12.0 software (SPSS, Chicago, IL, USA).

## 3 RESULTS AND DISCUSSION

### 3.1 Proximate composition of extruded meat analog at different mealworm larva content

The base formation of extruded meat analog was defatted soy protein (DSF), isolated soy protein (ISP), corn starch (CS), and mealworm larva. Major composition of these raw materials including moisture content, crude protein, and crude fat is shown in Table 1. DSF and ISP have high amount of protein content and have low crude fat content. But CS has the lowest crude protein content as 1.25%, which is generally used as binding agent in meat analog because it enhances cohesion among protein molecules (Asgar et al., 2010). Moreover, protein and starch interaction is also caused by consisting materials that will lead to Maillard reaction (Yilmaz & Toledo, 2005). According to the results, the plant meat analog consisting DSF, ISP, and CS have high content of crude protein whereas containing fat content was highly low. But mealworm lava revealed high content of 34.05% crude fat as well as 54.88% crude protein content. Therefore, it suggests that the addition of mealworm attribute to enhance the insufficient fatty acid content in plant meat analog and it could influence sensory properties such as texture and flavor (Cho & Ryu, 2017a; Van Huis, 2013).

**TABLE 1:** Proximate composition of raw material used to produce extruded meat analog

Proximate composition (%)			
Raw material	Moisture content	Crude protein	Crude fat
Mealworm larva	9.69 ± 0.25^1^ ^b,^ ^2^	54.88 ± 0.31^c^	34.05 ± 0.15^a^
Defatted soy flour	6.98 ± 0.32^c^	62.15 ± 0.15^b^	0.12 ± 0.09^b^
Isolated soy protein	6.75 ± 0.25^d^	92.44 ± 0.19^a^	0.8 ± 0.18^c^
Corn starch	11.5 ± 0.25^a^	1.25 ± 0.53^d^	0.6 ± 0.15^d^

^1^ Values are means of three replications ± standard deviation.

^2^ Means with different letters within a column are significantly different (*p* < .05) by Duncan's multiple range test.

### 3.2 TPA and WHC of extruded meat analog at different mealworm larva content

Texture profile analysis including hardness, cohesiveness, springiness, and chewiness of meat analog with addition of different mealworm contents are summarized in Table 2. The highest hardness, cohesiveness, springiness, and chewiness were observed in meat analog with 0% mealworm content extruded at 140°C die temperature and 50% moisture content (Table 2). Textural properties decreased with increase in mealworm content. This decrease was due to less texturization influenced by mealworm larva addition which led to weakening of internal molecular bonds. Similarly, Cho and Ryu (2017a) reported that hardness, cohesiveness, springiness, and chewiness of meat analog decreased with increased tuna sawdust content. Chiang et al. (2020) also reported that meat alternatives with 0% beef bone hydrolysate gave the highest hardness and chewiness. Therefore, the addition of animal protein could lead to poorer protein cross‐linking that resulted in softer texture compared to 100% extruded plant meat analog.

**TABLE 2:** Texture profile analysis (TPA) and water holding capacity (WHC) of extruded meat analog with different mealworm larva contents

Mealworm content (%)	Die temperature (°C)	Moisture content (%)	Hardness (g)	Cohesiveness (%)	Springiness (%)	Chewiness (g)	Water holding capacity (%)
0	140	40	4,560.21 ± 0.21^1^ ^b,^ ^2^	43.45 ± 0.05^b^	25.21 ± 0.05^b^	183.12 ± 0.05^b^	209.37 ± 0.09^k^
		50	5,214.32 ± 0.32^a^	48.45 ± 0.12^a^	26.37 ± 0.09^a^	213.90 ± 0.19^a^	211.21 ± 0.11^j^
	150	40	3,980.41 ± 0.96^d^	31.78 ± 0.09^d^	18.49 ± 0.21^d^	169.83 ± 0.09^d^	228.32 ± 0.19^i^
		50	4,168.32 ± 0.21^c^	35.42 ± 0.09^c^	21.54 ± 0.05^c^	172.14 ± 0.02^c^	231.99 ± 0.25^h^
15	140	40	2,982.34 ± 0.11^f^	11.01 ± 0.11^f^	15.43 ± 0.13^f^	94.90 ± 1.11^f^	257.21 ± 0.31^g^
		50	3,021.42 ± 0.81^e^	13.43 ± 0.05^e^	17.18 ± 0.02^e^	101.13 ± 0.02^e^	237.45 ± 0.09^f^
	150	40	2,210.43 ± 0.79^h^	9.54 ± 0.02^h^	13.45 ± 0.09^h^	84.50.±0.25^h^	258.09 ± 0.05^f^
		50	2,742.05 ± 0.21^g^	10.11 ± 0.05^g^	14.98 ± 0.12^g^	90.61 ± 0.12^g^	261.23 ± 0.12^e^
30	140	40	1802.34 ± 0.45^j^	0.94 ± 0.08^j^	1.66 ± 0.09^j^	20.19 ± 0.09^k^	298.01 ± 0.19^d^
		50	1954.32 ± 0.09^i^	1.59 ± 0.09^i^	2.45 ± 0.09^i^	38.98 ± 0.32^i^	261.43 ± 0.15^c^
	150	40	1,124.31 ± 0.13^l^	0.51 ± 0.15^l^	1.21 ± 0.05^l^	13.15 ± 0.16^j^	405.22 ± 0.35^b^
		50	1,780.79 ± 0.29^k^	0.83 ± 0.02^k^	1.32 ± 0.03^k^	18.72 ± 0.25^l^	421.78 ± 0.25^a^

^1^ Values are means of three replications ± standard deviation.

^2^ Means with different letters within a column are significantly different (*p* < .05) by Duncan's multiple range test.

On the other hand, the addition of plant materials such as green tea showed the increased trend in hardness and chewiness of meat analog with increase in green tea contents. The results were in opposite trend with those of animal protein addition. The increase in textural properties after green tea addition might be due to protein–polyphenol interaction which resulted in complex networking effect (Ma & Ryu, 2019).

Hardness, cohesiveness, springiness, and chewiness increased as die temperature decreased from 150 to 140°C and moisture increased from 40% to 50%. Lower die temperature and higher moisture content in extrusion process can inhibit the formation of porous and expanded structure and can enhance the fibrous structure formation of meat analog (Cho & Ryu, 2017a).

WHC is defined as the amount of water that can be bound to the meat analog structure after rehydration (Lin et al., 2002). Table 2 shows WHC of meat analog affected by different mealworm contents and extrusion process parameters. The highest value (4.21) was observed in meat analog with 30% mealworm larva extruded at 150°C die temperature and 50% moisture content. Previous study reported that WHC was highly affected by the porous structure of plant protein‐based meat analog (Samard & Ryu, 2019). Hence, the addition of mealworm larva resulted in the decreased denseness and weakened structure in extruded meat analog whereas it may be more favorable structure for holding moisture (Ma & Ryu, 2019).

Water holding capacity increased with the increased die temperature from 140°C to 150°C. Similar finding has been reported by Ma et al. (2018) who reported that WHC of green tea‐based extruded meat analog significantly increased with the increase in temperature. Therefore, high temperature affected the formation of porous structure of meat analog which led to high WHC.

### 3.3 Integrity index and NSI of extruded meat analog at different mealworm larva content

**FIGURE 1:**
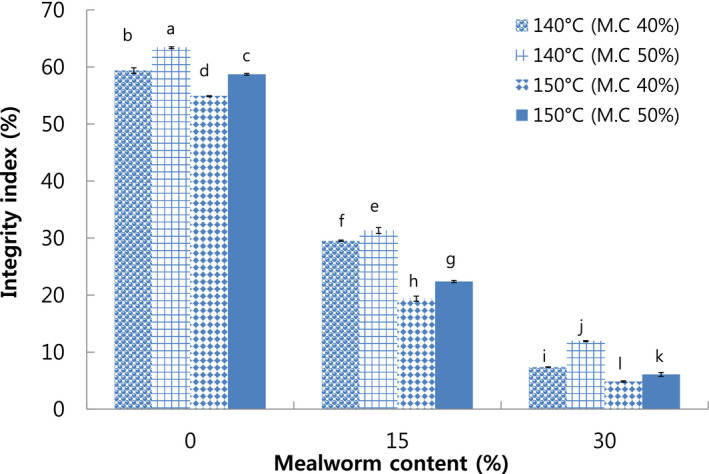
Integrity index of extruded meat analog with different mealworm larva contents. M.C: Moisture content. Different letters on the bars indicate significant differences (*p* < .05)

The integrity index refers to the residue texture after meat analog is hydrated, autoclaved, homogenized, and dried, which is an important property that indicates the quality of meat analog (Samard et al., 2019). The highest integrity index was 63.39% in meat analog with 0% mealworm larva content extruded at 140°C die temperature and 50% moisture content (Figure 1). The integrity index significantly decreased with the increase in the mealworm content and die temperature and the decrease in moisture content. This might be due to the weakened and dispersed structure of meat analog with high mealworm content extruded at higher die temperature and lower moisture content, which consequently led to a low integrity index.

**FIGURE 2:**
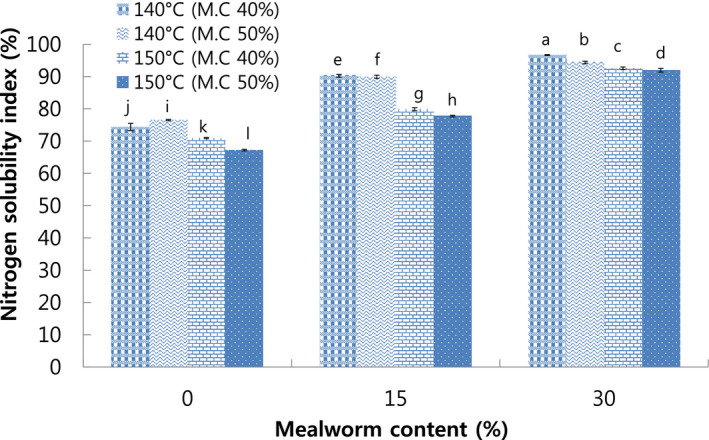
Nitrogen solubility index of extruded meat analog with different mealworm larva contents. M.C: Moisture content. Different letters on the bars indicate significant differences (*p* < .05)

Nitrogen solubility index was used to determine the soluble protein that was generated during the manufacture of meat analog (Samard et al., 2019). The highest (95.68%) was observed in meat analog with 30% mealworm larva content extruded at 140°C die temperature and 40% moisture content, while the lowest NSI (67.21%) was observed in meat analog with 0% mealworm content extruded at 150°C die temperature and 50% moisture content. The results showed that NSI increased with increase in mealworm larva content (Figure 2). Previous study reported that additive animal materials containing fat led to increased porous and weakened structure in extruded meat analog, which attributed to increased disperse and solubility in water (Cho & Ryu, 2017a). Therefore, the results revealed negative correlation among results of the integrity index which decreased with the increase in mealworm larva content (Figure 1). Furthermore, the increase in die temperature from 140°C to 150°C resulted in decreased NSI. It was due to the loss of solubility at higher die temperature that led to increased degree of protein denaturation (Cho & Ryu, 2017a).

### 3.4 PD of extruded meat analog at different mealworm larva content and comparison on total amino acids of raw mixture and extruded meat analog

Protein digestibility is essential for determining the nutritional value of food‐related research. However, there are few studies on digestibility and utilization of edible insects in human (Rumpold & Schlüter, 2015).

**FIGURE 3:**
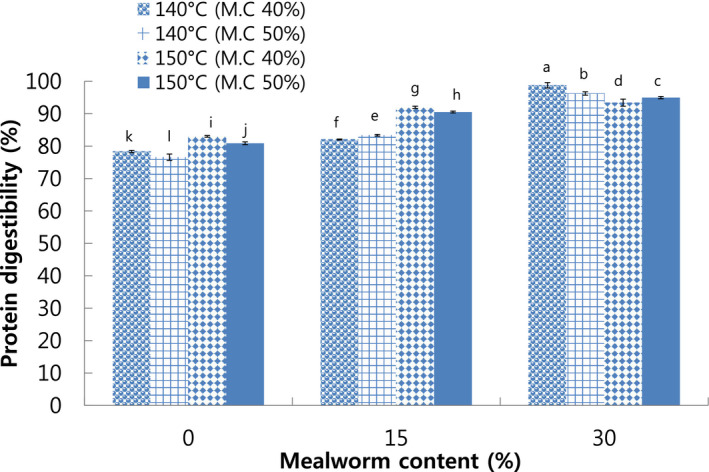
Protein digestibility of extruded meat analog with different mealworm larva contents. M.C: Moisture content. Different letters on the bars indicate significant differences (*p* < .05)

In the study, the highest PD (98.76%) was observed in meat analog with 30% mealworm larva extruded at 140°C die temperature and 40% moisture content, while the lowest PD (76.58%) was found in meat analog with 0% mealworm larva extruded at 150°C die temperature and 50% moisture content (Figure 3). PD values increased with increase in mealworm larva content. This result was in accordance with the pattern of nitrogen solubility index. Addition of mealworm larva improved the PD of extruded meat analog leading to increase in soluble amino acids absorbed by human body. High temperature and pressure during extrusion could remove the chitin from whole mealworm larva protein which might result in an increased digestibility (Song et al., 2018). Ozimek et al. (1985) reported that digestibility of whole honey bee without chitin removal was 71%. It suggests that chitin removal via high temperature (extrusion cooking) as well as alkaline extraction resulted in an increased digestibility of whole mealworm larva protein (Song et al., 2018).

The analysis of amino acids in extruded meat analog was performed before and after the extrusion cooking to investigate the effects of heat treatment and mealworm larva contents on the amino acid content of extruded meat analog. The samples with 30% mealworm larva content, at 140°C die temperature and 40% moisture content (fixed process parameters), were used for the analysis, due to its highest value in PD. In addition, 0% extruded meat analog with mealworm larva content, with same processing parameter, was used.

The highest total amino acid content was revealed as 591.43 g/kg in the raw mixture with 30% mealworm larva content before extrusion, while the lowest value was found as 499.03 g/kg in extruded meat analog with 0% mealworm larva (Table 3). The essential amino acid content decreased after extrusion. This was in accordance with the finding of Dias paes and Maga (2004) who reported that the essential amino acid levels of whole‐grain flour decreased after extrusion as the amino acids are susceptible to denaturation when exposed to high temperature during extrusion. However, sulfur‐containing amino acids such as methionine and cysteine, and glutamic acid were higher in extruded meat analog than in raw material mixture. Samard and Ryu (2019) reported that the increase in sulfur‐containing amino acids in extruded soy products was due to their heat stability during extrusion, while lysine was found to be the most unstable amino acid.

**TABLE 3:** Comparison of amino acids contents in raw mixtures and extruded meat analogs

Amino acids (g/kg^−1^ protein)	Raw		Extruded meat analog	
	0% mealworm	30% mealworm	0% mealworm	30% mealworm
Aspartic acid	60.90 ± 1.55^1^	64.53 ± 2.18	56.62 ± 0.12	63.07 ± 0.57
Threonine	21.71 ± 0.13	21.96 ± 0.34	20.62 ± 1.25	21.77 ± 0.16
Serine	27.52 ± 0.14	28.65 ± 1.25	25.63 ± 3.25	28.23 ± 2.13
Glutamic acid	85.34 ± 1.24	98.80 ± 4.25	93.80 ± 1.08	101.42 ± 3.42
Glycine	23.10 ± 0.16	23.25 ± 2.31	22.62 ± 0.35	22.74 ± 2.36
Alanine	22.72 ± 0.02	23.57 ± 0.17	23.73 ± 0.12	22.11 ± 0.45
Valine	26.11 ± 1.54	26.20 ± 0.24	25.21 ± 2.35	25.55 ± 1.89
Isoleucine	23.13 ± 1.24	24.14 ± 1.70	21.63 ± 1.25	23.54 ± 1.23
Leucine	38.73 ± 0.86	50.65 ± 3.51	35.99 ± 0.57	49.45 ± 2.34
Tyrosine	18.98 ± 0.15	33.04 ± 1.50	18.10 ± 2.60	30.63 ± 1.24
Phenylalanine	27.22 ± 0.26	49.25 ± 1.06	24.73 ± 0.06	47.88 ± 1.30
Lysine	33.25 ± 0.86	34.88 ± 1.50	31.22 ± 2.18	34.09 ± 0.85
Histidine	14.93 ± 0.15	28.39 ± 1.28	14.83 ± 1.25	22.15 ± 0.23
Arginine	38.65 ± 0.26	40.59 ± 1.24	34.44 ± 1.00	40.25 ± 0.34
Cystine	6.83 ± 1.28	8.91 ± 0.65	16.11 ± 1.24	14.38 ± 1.25
Methionine	6.95 ± 0.45	7.82 ± 2.61	8.22 ± 0.04	12.16 ± 0.56
Proline	26.66 ± 2.18	26.80 ± 0.04	25.53 ± 0.34	25.79 ± 4.45
Total contents	502.73	591.43	499.03	585.21

^1^ Values are means of three replications ± standard deviation.

The amino acid composition of soy protein consists of acidic amino acids such as aspartic and glutamic acids as major compounds and sulfur amino acids such as methionine and cysteine as minor ones (Kim, 2002). In the present study, glutamic acid was the highest among the amino acids present in extruded meat analog with 30% mealworm larva. Kim et al. (2003) reported that glutamic acid has a synergistic effect on flavor with the coexistence of other flavor compounds. The results of this study suggested that extrusion cooking with addition of mealworm larva could enhance the glutamic acid content which can influence the flavor improvement of meat analog. In addition, other amino acids including phenylalanine, tyrosine, and leucine also significantly increased in addition of 30% mealworm. Similar result was reported by Rumpold and Schlüter (2013) who stated that mealworm larva contains high amount of phenylalanine and tyrosine and generally meet the amino acid requirements except for methionine.

### 3.5 DPPH radical scavenging activity and TBARS of extruded meat analog at different mealworm larva content

Determination of DPPH radical scavenging activity which reflects the antioxidant content in foods is important for protection of deleterious changes and loss of commercial and nutritional values (Kedare & Singh, 2011).

**FIGURE 4:**
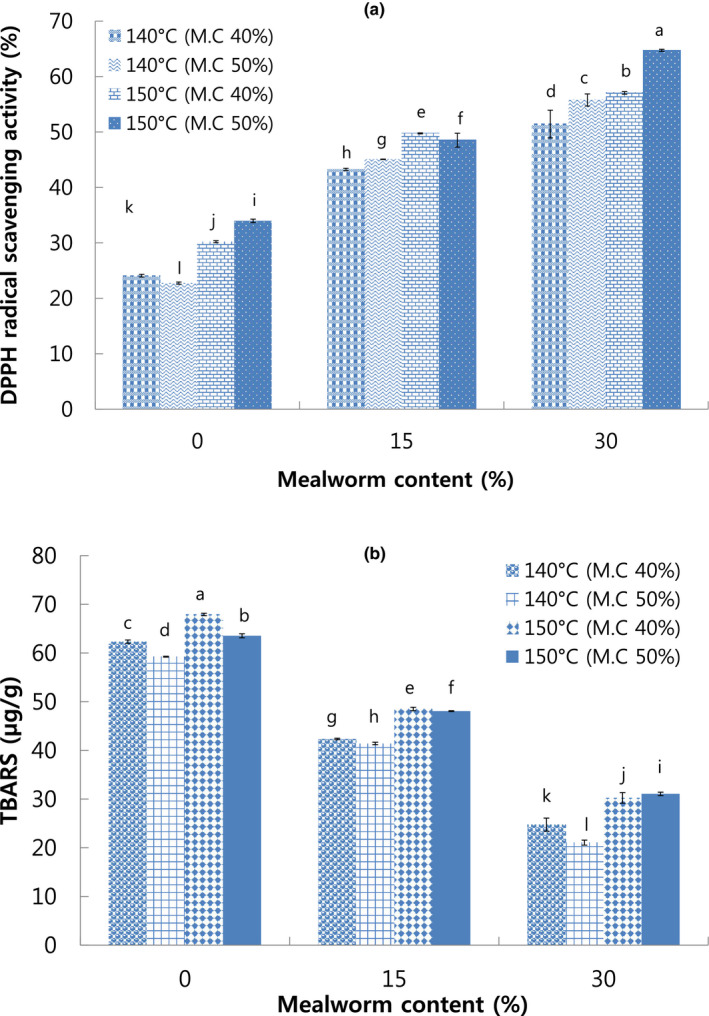
DPPH radical scavenging activity (a) and TBARS (2‐thiobarbituric acid reactive substances), (b) of extruded meat analog with different mealworm larva contents. M.C: Moisture content. Different letters on the bars indicate significant differences (*p* < .05)

In our study, the highest DPPH radical scavenging activity was 64.73% in meat analog with 30% mealworm larva content extruded at 150°C die temperature and 50% moisture content, while the lowest was 22.75% in meat analog with 0% mealworm larva content extruded at 140°C die temperature and 50% moisture content (Figure 4a). The DPPH radical scavenging activity significantly increased with the increase in mealworm larva content. This may be due to selenium or vitamin E in mealworm larva which can cause the decrease in levels of free radicals (Finke, 2002; Rumpold & Schlüter, 2013). These results were in accordance with the findings obtained by El‐Demerdash (2004). They reported that vitamin E and selenium could be beneficial in alleviating aluminum toxicity. Moreover, α‐tocopherol, which is a dominant form of vitamin E, or selenium has strong antioxidant functionality that terminates the self‐perpetuating cycle of lipid peroxidation on membrane‐bond.

In addition, DPPH radical scavenging activity increased with the increase in die temperature from 140°C to 150°C. Increase in DPPH activity might be related to natural phenolic substances such as isoflavone in soybeans that could be activated by heat treatment (Lee et al., 2014). Cho and Ryu (2017b) reported that DPPH radical scavenging activity of extruded rice snack significantly increased with increase in die temperature from 130°C to 140°C as well as increase in mealworm larva content (0, 10, and 20%). The increase in antioxidant activity is attributed to Maillard reaction in meat analog enhanced by high temperature since the Maillard reaction product possesses the antioxidant activity for scavenging the oxygen radicals or chelating metals (Song et al., 2013).

TBARS is a method using thiobarbituric acid (TBA) to detect lipid oxidation including malondialdehyde (MDA). The highest TBARS was 67.92 mg/kg in extruded meat analog with 0% mealworm larva extruded at 150°C die temperature and 40% moisture content, while the lowest was 21.05 mg/kg in meat analog with 30% mealworm larva extruded at 140°C die temperature and 50% moisture content. The TBARS value decreased with the increase in mealworm larva content. This might be related with the antioxidant compounds in mealworm larva, indicated by the high DPPH activity (Figure 4b), which could prevent the formation of lipid that occurred in extruded meat analog during process period (Cho & Ryu, 2017a; Rumpold & Schlüter, 2015).

Kim et al. (2009) also reported that there was an inverse correlation between antioxidant activity and peroxide value as shown in Figure 4a,b. Bozkurt and Erkmen (2002) similarly found that the sausages with addition of α‐tocopherol and ascorbic acid had the lowest TBARS values due to the protective effect of antioxidants on stability of oxidation in the sausage.

There was no correlation between TBARS values and moisture content. However, TBARS values increased as the die temperature increased from 140°C to 150°C (Figure 4b). This result suggested that higher temperature could lead to the increase in oxidation activity. It may be because of the rapid formation of oxidation substances from unstable lipids caused by high temperature during extrusion cooking. This finding was in accordance with Sun et al. (2020) who reported that the 2,4‐decadienal in oxidation indicator of edible oils increased with increase in heating temperature (120°C, 150°C, and 180°C).

### 3.6 Sensory evaluation of extruded meat analog

The sensory scores of extruded meat analogs with different mealworm larva contents are shown in Table 4. The highest overall acceptance score was observed in meat analog with 30% mealworm larva content extruded at 140°C and 50% moisture content. It was revealed that the scores for meaty taste, aroma, and overall acceptance increased with the increase in added mealworm larva content. These results were in accordance with the finding that the addition of 30% mealworm larva into the mixture had the highest glutamic acid level, which has synergistic effect on flavor with the coexistence of other flavor compounds (Table 3). However, the texture score decreased with the increase in mealworm larva content except for the sample extruded at 140°C and 50% moisture content. It may be due to the addition of mealworm larva which led to the softer texture and lower TPA values including hardness, cohesiveness, springiness, and chewiness (Table 2).

**TABLE 4:** Sensory evaluation of extruded meat analog with different mealworm larva contents

Mealworm content (%)	Die temperature (°C)	Moisture content (%)	Color	Texture	Meaty taste	Aroma	Overall acceptance
0	140	40	2.45 ± 0.63^1^ ^f,^ ^2^	2.62 ± 0.32^c^	1.25 ± 0.34^l^	1.25 ± 0.34^l^	1.91 ± 0.09^j^
		50	3.21 ± 0.94^d^	3.31 ± 0.48^a^	1.82 ± 0.94^j^	1.37 ± 0.69^k^	2.87 ± 0.63^e^
	150	40	1.91 ± 1.26^i^	2.81 ± 0.69^b^	1.37 ± 0.12^k^	2.01 ± 0.82^h^	1.25 ± 0.34^l^
		50	1.81 ± 0.25^j^	1.85 ± 0.70^f^	1.91 ± 0.07^i^	1.73 ± 0.70^j^	1.81 ± 0.51^k^
15	140	40	3.78 ± 0.11^b^	1.74 ± 0.11^g^	2.87 ± 0.31^f^	2.32 ± 0.84^g^	2.57 ± 0.31^f^
		50	4.46 ± 0.84^a^	2.10 ± 0.25^d^	1.98 ± 0.48^h^	1.91 ± 0.07^i^	1.98 ± 0.09^i^
	150	40	3.75 ± 0.69^c^	1.91 ± 0.07^e^	2.31 ± 0.03^g^	2.87 ± 0.32^e^	2.31 ± 0.48^g^
		50	1.75 ± 0.07^k^	1.64 ± 0.94^i^	3.45 ± 0.70^e^	2.62 ± 0.93^f^	2.01 ± 1.89^h^
30	140	40	1.64 ± 0.31^l^	1.59 ± 0.08^j^	3.68 ± 0.82^d^	4.31 ± 0.84^b^	3.87 ± 0.51^c^
		50	2.81 ± 0.37^e^	1.72 ± 0.15^h^	4.46 ± 0.84^a^	3.57 ± 0.81^d^	4.59 ± 0.50^a^
	150	40	2.01 ± 0.89^g^	1.25 ± 0.34^l^	4.01 ± 0.51^c^	4.68 ± 0.16^a^	3.71 ± 0.12^d^
		50	1.98 ± 0.09^h^	1.40 ± 0.75^k^	4.34 ± 0.23^b^	3.98 ± 0.25^c^	4.01 ± 0.25^b^

^1^ Values are means of three replications ± standard deviation.

^2^ Means with different letters a column are significantly different (*p* < .05) by Duncan's multiple range test.

The highest color score was obtained in meat analog with 15% mealworm content and extruded at 140°C and 50% moisture content. The texture score increased as die temperature decreased from 150°C to 140°C, and moisture content increased from 40% to 50%. According to this finding in combination with those of TPA, integrity index, and WHC, it suggested that the consumers would potentially prefer the meat analog with fibrous meat‐like texture. Overall, the optimal sensory level of extruded meat analog was in 30% mealworm larva addition, extruded at 150°C and 50% moisture content.

## 4 CONCLUSION

In this study, the addition of mealworm larva enhanced protein solubility and digestibility (NSI, PD) and total amino acids as well as antioxidant activity whereas weakened the texturization of extruded meat analog. Extrusion process parameters including die temperature (140°C and 150°C) and moisture content (40% and 50%) also influenced the textural and chemical properties of extruded meat analog.

Addition of mealworm larva led to the decrease in hardness, cohesiveness, springiness, and chewiness and the increase in NSI attributed to the weakening of internal molecular bonds and poorer cross‐linking in extruded meat analog. Meanwhile, the meat analog with mealworm possessed the structure favorable for holding moisture resulting in increased WHC. Among extrusion process parameters, higher die temperature (150°C) and lower moisture (40%) inhibited the fibrous structure formation which reduced the textural properties of meat analog.

Protein digestibility and total amino acids increased after addition of mealworm larva whereas subsequent denaturation by high temperature during extrusion led to the decrease. After extrusion, the glutamic acid which is a flavor‐enhancing amino acid was found to be the most abundant and it was highest in meat analog with 30% mealworm larva. The DPPH radical scavenging activity significantly increased after addition of mealworm. There was an inverse relationship observed between DPPH activity and lipid peroxidation. Addition of mealworm larva enhanced the antioxidant activity and reduced the oxidation. Moreover, Maillard reaction via extrusion temperature had effect on the increase of antioxidant property.

Overall, although the addition of mealworm larva weakened the texture of meat analog, it could enhance the nutritional and antioxidant properties and flavor of extruded meat analog under specific extrusion process conditions.

## CONFLICT OF INTEREST

The authors declare that they have no conflict of interest.

## ETHICAL APPROVAL

This study does not involve any human or animal testing.
